# Investigating AD and non-AD [^18^F]flortaucipir distribution patterns in a memory clinic cohort

**DOI:** 10.1007/s00259-026-07995-z

**Published:** 2026-06-15

**Authors:** Francesco Piva, Cecilia Boccalini, Nicholas J. Ashton, Henrik Zetterberg, Kaj Blennow, Giovanni B. Frisoni, Mattia Veronese, Valentina Garibotto, Débora Elisa Peretti

**Affiliations:** 1 Department of Information Engineering, University of Padua, Padua, Italy; 2 Laboratory of Neuroimaging and Innovative Molecular Tracers, University of Geneva, Rue Gabrielle-Perret-Gentil 4, 1205 Geneva, Switzerland; 3 Centre for Age-Related Medicine, Stavanger University Hospital, Stravanger, Norway; 4 Department of Psychiatry and Neurochemistry, The Sahlgrenska Academy at the University of Gothenburg, Mölndal, Sweden; 5 Institute of Psychiatry, Psychology & Neuroscience, King-S College London, London, UK; 6 NIR Biomedical Research Centre for Mental Health & Biomedical Research Unit for Dementia at South London & Maudsley NHS Foundation, London, UK; 7 Dementia Research Institute, University College London, London, UK; 8 Department of Neurodegenerative Diseases, University College London, London, UK; 9 Clinical Neurochemistry Laboratory, Sahlgrenska University Hospital, Mölndal, Sweden; 10 Department of Pathology and Laboratory Medicine, University of Wisconsin School of Medicine and Public Health, Madison, WI USA; 11 Wisconsin Alzheimer’s Disease Research Centre, University of Wisconsin-Madison, Madison, USA; 12 Paris Brain Institute, Sorbonne University, Paris, France; 13 Neurodegenerative Disorder Research Centre, University of Wisconsin-Madison, Madison, USA; 14 Laboratory of Neuroimaging of Aging, University of Geneva, Geneva, Switzerland; 15 Geneva Memory Center, Geneva University Hospitals, Geneva, Switzerland; 16 Department of Neuroimaging, King’s College London, London, UK; 17 Division of Nuclear Medicine and Molecular Imaging, Geneva University Hospitals, Geneva, Switzerland; 18 Centre for Biomedical Imaging, University of Geneva, Geneva, Switzerland

**Keywords:** Alzheimer’s disease, [^18^F]flortaucipir, PET, SSM/PCA, Plasma biomarkers

## Abstract

**Purpose:**

Amyloid (A) deposition represents a specific pathological hallmark of Alzheimer’s disease (AD). Clinical diagnostic protocols frequently rely on the combined use of amyloid (A)- and tau (T)- positron emission tomography (PET) imaging to distinguish AD from non-amyloid-associated neurodegenerative conditions. Many neurodegenerative disorders are characterized by distinct tauopathies, which result in different [^1^⁸F]flortaucipir T-PET topographic patterns. The aim of this study was to investigate, in a memory clinic cohort, whether [^18^F]flortaucipir distribution patterns derived from scaled subprofile modelling using principal component analysis (SSM/PCA) could differentiate between A + and A − individuals, potentially eliminating the need for an A-PET.

**Methods:**

A total of 81 subjects were included in this study: 40 with AD characterized by both A and T accumulation (A + T +), 13 with suspected non-Alzheimer’s pathology (A − T +), and 28 without A and T abnormality (A − T −). SSM/PCA was applied on [^18^F]flortaucipir images to identify two disease patterns (DPs), representing amyloid-positive (A + DP) and amyloid-negative (A– DP) accumulation. Each subject’s imaging data was then compared to these DPs and the resulting similarity scores were used as inputs for three machine learning models – Support Vector Machine, Random Forest, and Multi-Layer Perceptron – to predict amyloid status. Finally, Spearman correlation analyses were conducted between SSM/PCA-derived scores and plasma biomarkers levels.

**Results:**

The two identified disease patterns exhibited distinct PET signatures consistent with current disease knowledge. Their representativeness was further supported by significant correlations with plasma biomarkers in nearly all cases. All machine learning models confirmed the capacity of the A + DP to predict AD pathology, correctly classifying at least 36 out of 40 cases. In contrast, the A − DP showed limited discriminative power for non-AD conditions, as indicated by its higher misclassification rate (best performance: 7 correct out of 13).

**Conclusion:**

SSM/PCA applied on [^18^F]flortaucipir-PET imaging provide information on both A and T status, supporting its potential use as first and possibly sole examination in the investigation of suspected AD. While for non-AD cases, A-PET scans are still recommended.

**Supplementary Information:**

The online version contains supplementary material available at https://doi.org/10.1007/s00259-026-07995-z.

## Introduction

Alzheimer’s disease (AD) is a progressive neurodegenerative disorder that leads to cognitive decline. It is currently the most common cause of dementia worldwide, and due to the global aging of the population, its prevalence is expected to rise significantly in the coming years [1, 2]. AD is primarily characterized by two pathological hallmarks: the extracellular deposition of amyloid (A) plaques and the intracellular accumulation of neurofibrillary tau (T) tangles [3]. Notably, A deposition begins many years before the onset of clinical symptoms, making it one of the earliest detectable makers of AD pathology [4]. In contrast, recent studies have shown that T pathology is more closely associated with cognitive decline and symptom onset, making it a stronger predictor of disease progression [5, 6].

Both A and T biomarkers can be expressed in a binary form, classifying individuals as either as positive or negative [7].

[^18^F]flortaucipir-PET was developed to detect AD tau pathology, showing cortical binding patterns, consistent with the neuropathological Braak stage above or equal to IV, extending beyond the medial temporal/fusiform area (e.g., with lateral temporal, parietal, or frontal cortex binding) [8]. Neurodegenerative diseases not classified as AD are typically characterized by the absence of A deposition [3] and by a different form of tauopathy, although co-pathology frequently occur. While [^18^F]flortaucipir-PET has demonstrated excellent correlation with neurofibrillary tangles distribution in AD, its utility in non-AD pathology remain debated [9]. Indeed, its selectivity is not well established with PET-to-autopsy comparisons suggesting that [^18^F]flortaucipir-PET had poor sensitivity and specificity for non-AD tauopathies [10]. Nevertheless, recent studies have reported increased cortical and subcortical binding in non-AD conditions such as frontotemporal lobar degeneration (FTLD) [11], and significant in vivo binding in carriers of specific MAPT mutations known to engender tau pathology [12–14].

Given this context, clinical protocols often rely on the combination of A- and T-PET scans to differentiate AD patients from those with other neurodegenerative conditions. However, a diagnostic approach based on a single PET scan capable of capturing both aspects of the disease would offer substantial practical advantages. A study published by Hammes and colleagues already explored this possibility, showing that scaled subprofile modelling using principal component analysis (SSM/PCA) applied to [^18^F]flortaucipir data could provide information on both A and T pathology [14].

SSM/PCA is a multivariate voxel-based technique that identifies disease-specific topographic patterns by comparing patient scans to a reference group free of pathology [15]. Once a disease pattern (DP) has been identified, it can be used to evaluate new subjects. The resulting score reflects the similarity between a subject’s scan and the DP, serving as an indirect measure of disease expression. Moreover, this score can be correlated with other markers of disease severity, such as neuropsychological assessments or fluid biomarkers. Significant correlations would support the validity of the DP as representative of the underlying pathology and confirm the subject-specific expression scores as quantitative biomarkers of disease progression. Recent applications of SSM/PCA have successfully identified metabolic patterns in neurodegenerative diseases such as AD and Parkinson’s disease using ^18^F-FDG PET [16, 17]. The potential to use a single PET tracer to characterize multiple aspects of a disease is gaining interest, as multi-tracer protocols are more costly, increase patient burden, and elevate radiation exposure [17].

In this study, we investigated, in a memory clinic cohort, whether [^1^⁸F]flortaucipir DPs derived from SSM/PCA could differentiate A + from A − individuals, thereby offering a clinically useful approach to streamline the differential diagnosis of AD from other neurodegenerative conditions.

## Material and methods

### Study cohort

A cohort of 81 subjects was selected from an ongoing study at the Geneva Memory Clinic at the Geneva University Hospitals (HUG), Geneva, Switzerland. These subjects were selected from a larger cohort of available subjects since they fitted the following inclusion criteria: (1) A and T-PET imaging performed within 12 months of each other; (2) T1-weighted magnetic resonance imaging (T1w-MRI) scan performed within 6 months of PET imaging.

This memory clinic cohort included subjects ranging from cognitive unimpaired (CU), over mild cognitive impairment (MCI), to dementia, or other mental disorders. Diagnosis was based on a clinical assessment combined with the results of the neuropsychological assessment, which also included a Mini-Mental Sate Examination (MMSE).

The local review board (Commission cantonale d’éthique de la recherche – CCER de Genève) approved the study, which was conducted in concordance with the principles of the Declaration of Helsinki and the Internation Conference on Harmonization Good Clinical Practice. Each subject provided written informed consent for participation in the study.

### Image acquisition and processing

All PET scans using [^18^F]flortaucipir were performed at the Division of Nuclear Medicine and Molecular Imaging at Geneva University Hospitals with Biograph128 mCT, Biograph128 Vision 600 Edge, Biograph40 mCT, or Biograph64 TruePoint PET scanners (Siemens Medical Solutions, Malvern, PA, USA). All scanners were from the same vendor and generation and were harmonized in terms of performance and reconstruction parameters, with cross-calibration procedures regularly performed across scanners. The harmonisation between scans was performed using EARL guidelines as the HUG is an EARL-certified centre. The images used in this study were acquired before the certification. However, images comply to the the criteria of spatial resolution based on the PET/CT scanner model and reconstruction parameters. A-PET images were acquired using either [^18^F]florbetapir or [^18^F]flutemetamol. For [^18^F]florbetapir, images were acquired 40 min after intravenous administration of 209 ± 21 MBq for 10-min acquisition. Tau-PET scans were performed using [18F]flortaucipir. Subjects received 202 ± 48 MBq of [18F]flortaucipir, and image acquisition started 75 min after injection (acquisition time: 30 min). For all tracers, data were acquired in list mode and reconstructed using 3D ordered subset expectation (OSEM). The reconstruction process included corrections for randoms, dead time, normalization, scatter, attenuation, and sensitivity. Motion correction was applied, followed by spatial smoothing using a 2-mm full-width at half-maximum (FWHM) Gaussian filter. The reconstructed images had a matrix size of 400 × 400 with isotropic voxels of 1.01 mm. PET processing was performed using Statistical Parametric Mapping (SPM12, Wellcome Trust Centre for Neuroimaging, London, UK), running in MATLAB R2023b Version 9.5 (MathWorks Inc., Sherborn, MA, USA). All PET images were co-registered to the subjects respective T1w-MRI scan and normalized to the Montreal Neurologic Institute (MNI) standard space by applying the transformation parameters obtained from the MRI normalization.

### Plasma sampling and processing

Plasma samples were collected within a year of T-PET examination, with participants non-fasting. Blood was collected in EDTA-plasma tubes and centrifuged (2000 g, + 4^*◦*^C for 10 min). Following centrifugation, plasma was aliquoted into 1.5 ml polypropylene tubes (1 ml plasma in each tube) and stored at −80^*◦*^C in polypropylene tubes. A-beta(β) _42_ and Aβ _40_ ratio (Aβ _42_/Aβ _40_), phosphorylated-tau (p-tau) 181, neurofilament light (NfL) and glial fibrillary acidic protein (GFAP) concentrations were measured using Single molecule array technology and commercially available kits on an HD-X instrument (Quanterix, Billerica, MA) [18]. P-tau231 concentration was measured using an in-house Simoa assay on an HD-X instrument (Quanterix Billerica, MA), as previously described [19].

### AT profiles computation

AT profiles were computed considering semi-quantitative measures based on PET images. *A*: standardized uptake value ratio (SUVR) was computed using the whole cerebellum as reference region. SUVR was derived from the Centiloid (CL) region of interest (ROI) and transformed into CL units, following the procedure by Klunk and colleague [20]. A CL threshold of 19 was employed to distinguish between A − and A + [21]. *T*: SUVR was computed using the cerebellar crus as a reference region. Global SUVR was considered, which was calculated from the parahippocampal gyrus, middle occipital cortex, inferior temporal cortex and amygdala [22]. A global SUVR cut-off point of 1.24 was used to distinguish between T + and T-[23]. This process yields a unique profile for each subject, which can be classified into one of four potential categories: A − T −, A − T +, A + T −, A + T +, where:

- *A* − *T* − *group,* consisting of 28 subjects, who do not exhibit AD-related pathophysiological changes, but may be healthy-aged controls or affected by other neurodegenerative or psychiatric conditions.
- *A* − *T* + *group*, composed of 13 subjects, suspected to reflect non-AD pathology.
- *A* + *T* − group, composed of 17 subjects suspected to be in Alzheimer’s neuropathologic change.
- *A* + *T* + *group*, comprising 40 subjects with AD pathology.

A + T − cover part of the analysis, as it was used to test the negative predictive value of computed DPs. To facilitate evaluation of the applied thresholds, the distributions of the amyloid and tau PET measures are presented as histograms for each group, including clinical diagnosis (Supplementary Figure S1).

### Statistical analysis

Continuous variables were assessed for normality using the Shapiro–Wilk test within each group. Based on the assessment of normality, Kruskal–Wallis tests were performed to compare age, years of education, MMSE score, global and regional tau SUVR, and CL values across groups, and plasma biomarkers levels (the latter analysed only in participants with available plasma samples, *n* = 43) between groups. When the Kruskal–Wallis test was significant, post hoc pairwise comparisons were conducted using Dunn’s test with Bonferroni-Holm adjusted *p*-values. Chi-squared tests were performed for the gender variable. A two-sided *p*-value < 0.05 was considered statistically significant. Data were analysed using Python 3.11.5 (CPython; macOS 64-bit; Python Software Foundation; 24 Aug 2023).

### Univariate voxel-wise analysis

Voxel-wise two-sample t-tests were conducted to compare [^18^F]flortaucipir PET images between each pair of groups (i.e., A − T − vs. A + T +; A − T − vs. A − T +; A − T + vs. A + T +). No additional smoothing was applied, and all analyses were restricted to a whole-brain mask. SUVR images, which were already spatially normalized, were used, with no proportional scaling or other intensity normalization applied. T-maps were investigated at the voxel level using an uncorrected *p* = 0.001, and then a family-wise error (FWE) correction at cluster level was applied to select significant clusters of voxels. Analyses were performed using SPM12. Thresholded SPM images were overimposed on a standard CH2 brain with cerebellum surface using BrainNet Viewer [24].

### Multivariate analysis: Scaled subprofile modelling using principal components analysis

In the first phase, SSM/PCA was applied on all [^18^F]flortaucipir-raw images to explore data variance. In the second phase, SSM/PCA was performed separately within subgroups consisting of either A − T − and A + T + subjects or A − T − and A − T + subjects, aiming to identify areas of highest variance that characterise each subgroup. This led to the generation of two [^18^F]flortaucipir DPs, termed A + and A − DP, reflecting amyloid-positive and amyloid-negative neuropathology respectively. In both cases, the omitted group and A + T − subjects served as test set.

SSM/PCA was applied using an in-house software based on the work of Spetsieris and colleagues [25]. Initially, an external whole-brain mask, including the ventricles, was applied to [^18^F]flortaucipir PET raw images to exclude non-brain voxels and off-target regions such as the eyes and pituitary gland. A log transformation was then applied. Following the SSM procedure, subject-wise mean centering was first applied to remove global subject effects, and subsequently voxel-wise mean centering was performed by subtracting the voxel-wise mean calculated across the A − T − group, which served as the reference group. PCA was then applied to the centered data, yielding a set of principal components (PCs) ordered by explained variance. Component scores were subsequently standardized to Z-scores using the mean and standard deviation of the A − T − group as reference, enabling comparison across subjects and groups. Each subject received a score for each PC via inner product calculation, reflecting the expression of each PC in their PET scan. The first phase, termed “1D analyses”, extracted only the first component (accounting for the greatest variability) and its corresponding scores. Group comparisons of these scores were performed using the Kruskal–Wallis test, followed by Dunn’s post hoc test with Bonferroni Holm-adjusted p-values to correct for multiple comparisons. In the second phase, PCs collectively explaining at least 50% of the data variance were selected. A stepwise logistic regression was then employed to identify the most discriminative scores based on the lowest Akaike information criteria (AIC). The selected PCs were combined using model-derived weights to construct the DP. A leave one-out cross-validation (LOOCV) approach was used to assess the stability of scores of training subjects. Each omitted subject received a score independent of the pattern identification, minimizing bias. The generated DP was subsequently evaluated in the independent test subjects, corresponding to subjects not used for pattern derivation, and each subject received a corresponding pattern expression score. This procedure was repeated twice, for the computation of the A + and A − DP s. Consequently, each subject’s image received two scores (one for each DP), forming the basis of the “2D analyses.”

In both 1D and 2D analyses, scores were standardized to a Z-score using the A-T- group's mean and standard deviation as reference. Group comparisons were conducted using the Kruskal–Wallis test with Dunn’s post-hoc test and Holm correction for multiple comparisons. To assess the contribution of demographic and clinical covariates to the scores, a linear model (score ~ group + age + sex + education + MMSE) was fitted. Furthermore, Spearman correlation between the scores and continuous biomarker (CL and global tau SUVR values) were also assessed. All analyses were implemented in Python 3.11.5 (CPython; macOS 64-bit; Python Software Foundation; 24 Aug 2023).

### Amyloid status prediction

From the SSM/PCA step, two datasets were generated. In the 1D analysis, the subject scores derived from the first principal component (PC1) were used as a single continuous predictor to assess their ability to discriminate amyloid status. The A + T + group was defined as the positive class, while the A − T + and A − T − groups were considered as the negative class. A receiver operating characteristic (ROC) analysis was performed and the optimal classification threshold was determined by maximizing Youden’s index (sensitivity + specificity − 1) [26].

In the 2D analysis, each subject’s image was assigned two scores. The input dataset was visualised in a scatter plot to illustrate the distribution of these features. Three classification models – support vector machine (SVM), random forest (RF), and multilayer perceptron (MLP) – were tested to predict amyloid status. For each model, optimal hyperparameters were identified through grid search within stratified k-fold cross-validation framework. Subsequently, the final model was evaluated using cross-validation. Multiple metrics, including macro-F1, weighted-F1, and balanced accuracy, were computed to account for potential class imbalance and to provide a comprehensive performance assessment. ROC curves and AUC values were also calculated based on these cross-validation predictions. To assess the robustness of the models to class imbalance, a sensitivity analysis was performed by randomly undersampling the larger classes to match the size of the smallest class. This procedure was repeated 30 times to estimate variability and approximate confidence intervals. Among the three classifiers, the best-performing model was selected based on these metrics. All analysis were performed using Python 3.11.5 (CPython; macOS 64‑bit; Python Software Foundation; 24 Aug 2023).

### Correlation between plasma biomarkers and SSM/PCA scores

Spearman correlation was conducted to assess potential associations between plasma biomarkers and 2D scores, yielding a correlation coefficient (*rs*). Given the sample size, and deviations from normality of some variables, non-parametric rank correlation was retained more robust.A *p*-value below 0.05 was considered statistically significant. For a better visualisation, a scatter plot was generated for each variables pair. Data were analysed using Python 3.11.5 (CPython; macOS 64‑bit; Python Software Foundation; 24 Aug 2023).

## Results

### Study cohort

Demographic and clinical characteristics of the study cohort are summarized in Table 1. A statistical difference in age (*p* < 0.05) and MMSE score (*p* < 0.01) was observed between groups. Post hoc Dunn’s tests revealed that the MMSE difference was significant between the A − T − and A + T + groups (*p* < 0.01), while the age difference was significant between the A − T − and A + T − groups (*p* < 0.05). No significant differences were found with respect to gender and education years between groups.

**Table 1:** Characteristics of the study cohort. No significance difference was found for the included variables, except for MMSE. Superscript letters indicate groups showing significant differences at post-hoc comparisons: a > b

Group name	A − T − (*n* = 28)	A + T − (*n* = 17)	A − T + (*n* = 13)	A + T + (*n* = 40)	*p*	${\eta }^{2}$
Cognitive stage	CU (*n* = 14) MCI (*n* = 12) Other mental disorders (*n* = 2)	SCD(*n* = 3) MCI (*n* = 13) Dementia (*n* = 1)	CU (*n* = 4) MCI (*n* = 5) Dementia (*n* = 1) Other mental disorders (*n* = 3)	MCI (*n* = 25) Dementia (*n* = 15)	-	-
Age (years)	72 (67–75)^b^	78 (73–80)^a^	75 (74–77)	76 (71–79)	< 0.05	0.08
Gender (M/F)	16/12	11/6	5/8	18/22	0.46	0.14
Education (years)	14 (12–17)	13 (11–17)	15 (12–17)	12 (9–15)	0.16	0.02
MMSE	28 (27–29)^a^	27 (25–29)	26 (24–28)	25 (21–27)^b^	< 0.01	0.17

### [^18^F]flortaucipir comparison between groups

**Fig. 1:**
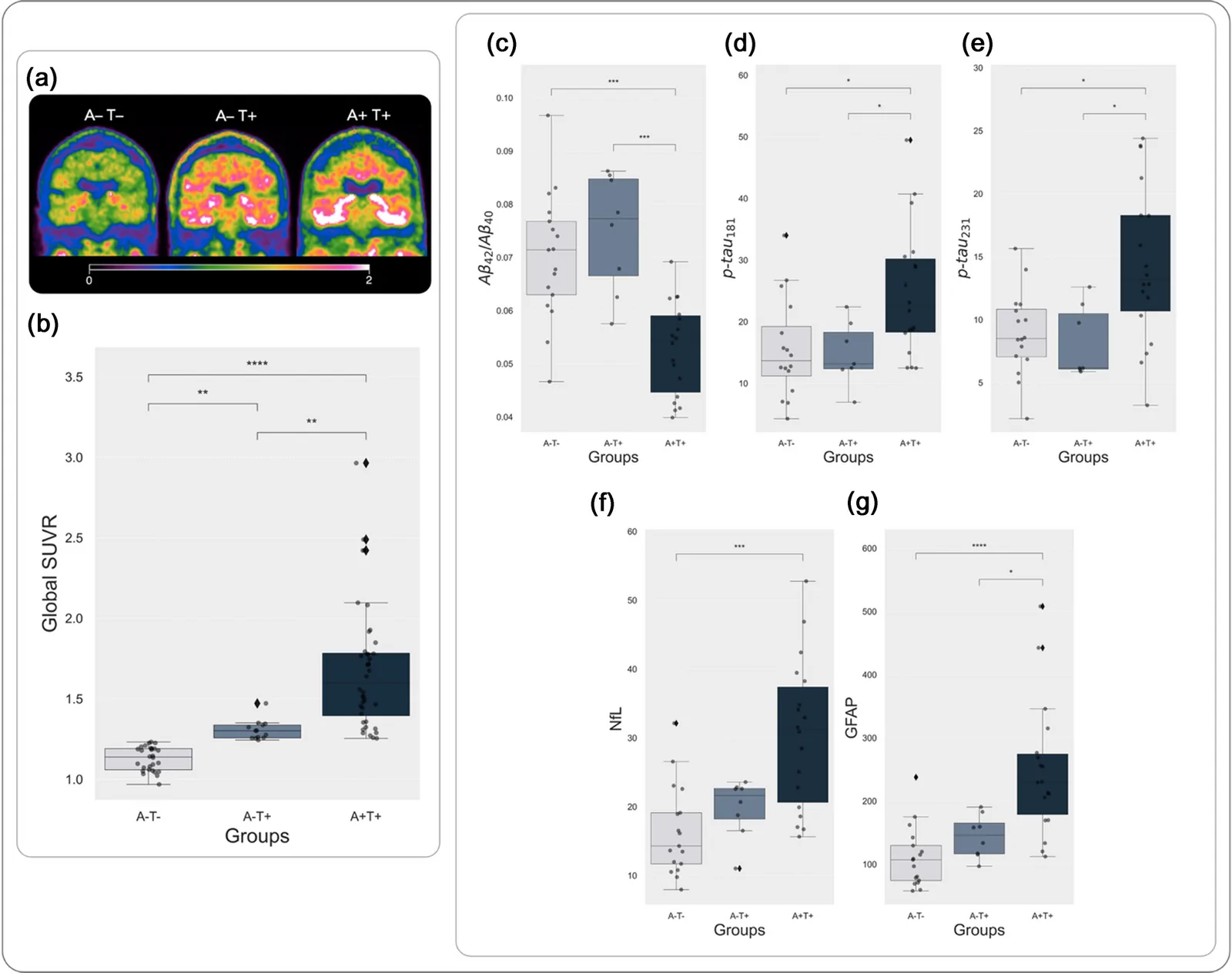
(**a**) [^18^F]flortaucipir binding (SUVR) in one representative case of each group. (**b**) Global [^18^F]flortaucipir SUVR comparison between groups. (**c**-**g**) plasma biomarkers comparison. * indicates significant p-values and corresponds post-hoc tests following significant Kruskal–Wallis test. *, 0.01 < *p* ≤ 0.05; **, 0.001 < *p* ≤ 0.01; ***, 0.0001 < *p* ≤ 0.001; ****, *p* ≤ 0.0001. Abbreviations: SUVR, standardized uptake value ratio; PC, principal component, Aβ, amyloid-beta; p-tau, phosphorylated-tau; NfL, neurofilament light; GFAP, glial fibrillary acidic protein

Figure 1a illustrates [^18^F]flortaucipir binding in three representative individuals from the A–T–, A–T +, and A + T + groups. To highlight the signal in the A–T + participant, we selected a slice centered on the medial temporal lobe, a region typically involved in the earliest stages of tau pathology. As expected, the A + T + individual shows elevated [^1^⁸F]flortaucipir uptake compared with the A–T– and A–T + cases, the latter displaying an intermediate pattern consistent with early tau involvement.

When examining the global SUVR values (Fig. 1b), a significant difference between groups was observed, both overall and after correction for multiple comparisons. Regional comparisons are presented in Supplementary Figure S2 and show consistent results.

To complement tau PET analyses, comparisons of CL values across groups are reported in Supplementary Figure S3.

### Plasma biomarkers comparison between groups

For each plasma biomarker, a significant overall difference was observed (*p* < 0.01,). After correction for multiple comparisons, significant differences were found between A − T − and A + T +, and between A − T + and A + T + groups for all biomarkers except for NfL (Fig. 1c-g). No statistically significant differences were found between the A − T − and A − T + groups for any biomarker.

### Univariate voxel-wise analysis

**Fig. 2:**
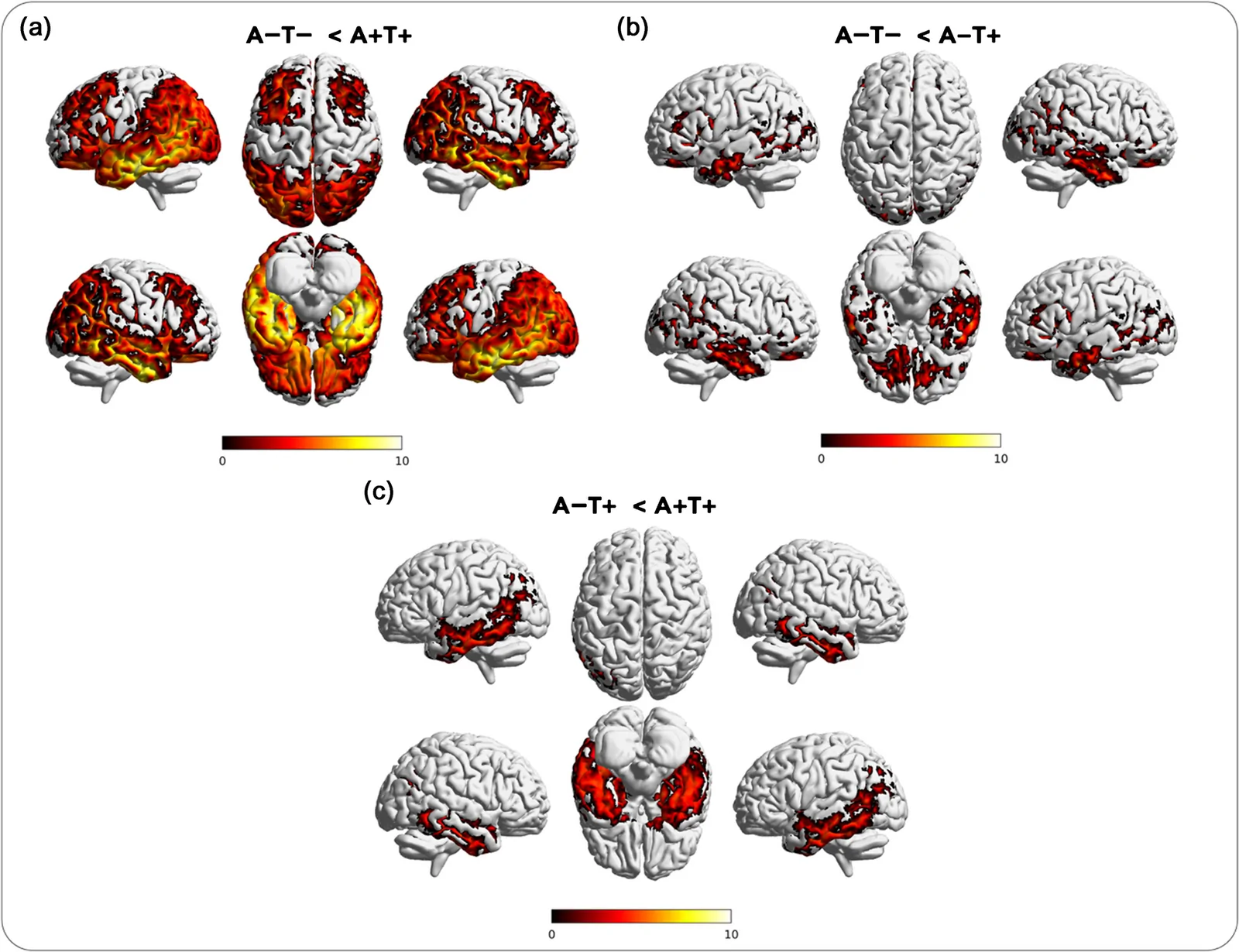
Two-sample *t*-test statistical parametric mapping (SPM) axial maps rendered onto a normalized T1w MRI image showing areas of significant differences. The colour intensity corresponds to the magnitude of the statistical difference observed. Thresholded SPM images (*p* < 0.001, FWE-corrected at the cluster level) are overimposed on a standard CH2 brain with cerebellum surface using BrainNet Viewer. (**a**) A − T − < A + T +, (**b**) A − T − < A − T +, (**c**) A − T + < A + T +

No significant differences were observed in the comparisons of A − T − > A + T + and A − T − > A − T +. Only a few isolated voxels in the cerebellum showed differences in the A + T + < A − T + contrast (available on Supplementary Figure S4). Consequently, three of the six maps generated from the voxel-wise *t*-test are presented in Fig. 2.

Compared to the A − T −, A + T + patients exhibited a significant increase in [^18^F]flortaucipir uptake across widespread cortical regions, with the strongest differences located in parietotemporal and frontolateral areas (Fig. 2a). In the A − T + group, the comparison with A − T − group revealed scattered clusters of voxels through the brain, including temporal regions (Fig. 2b). The voxel-wise t-test for the A − T + < A + T + contrast (Fig. 2c) showed statistically significant bilateral clusters in the temporoparietal cortex, with a predominance in the temporal lobe.

### Multivariate analysis: SSM/PCA

**Fig. 3:**
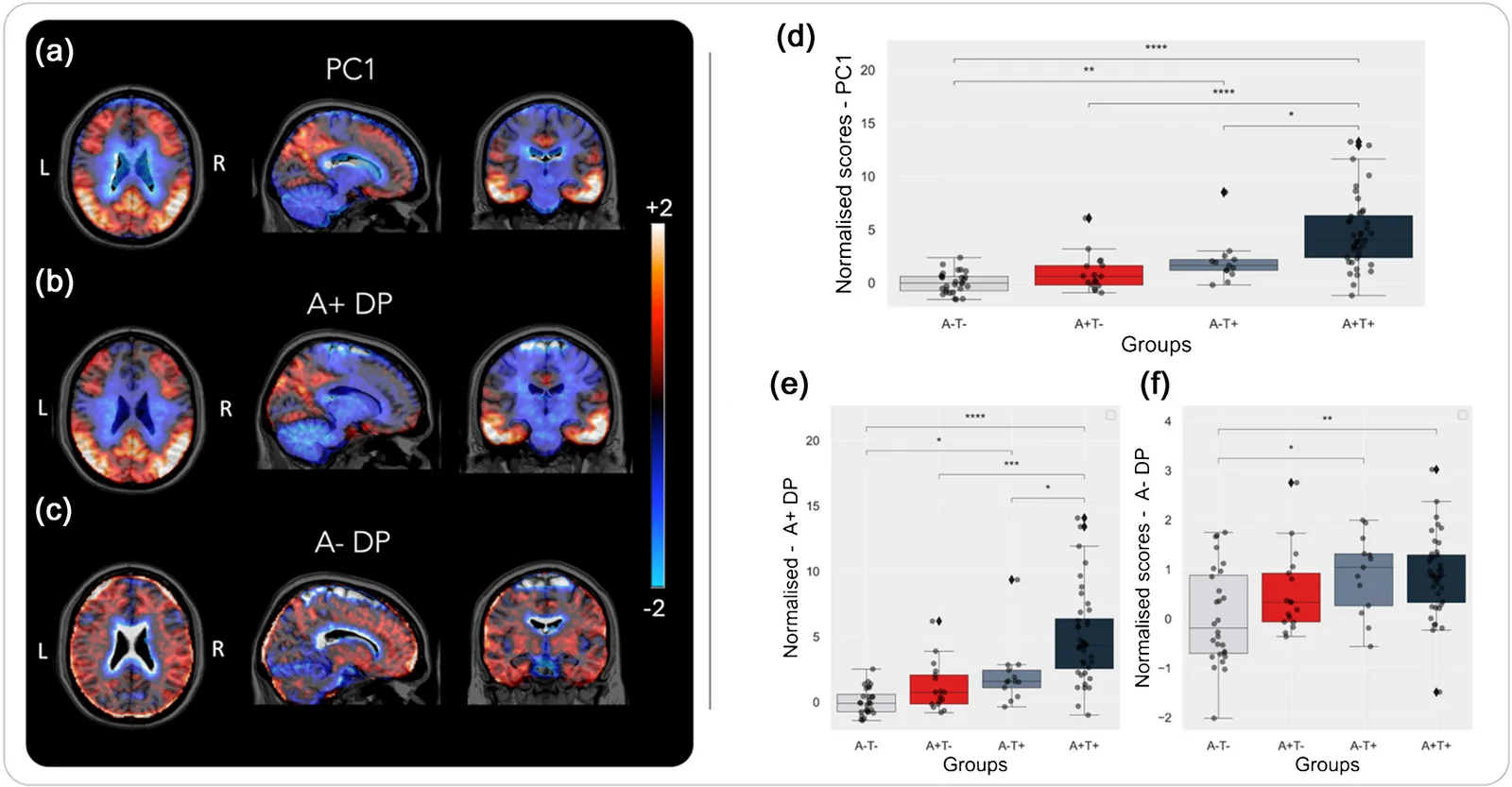
(**a**) PC1 computed combining all study participant together. (**b**) A + DP computed combining A-T- and A + T + groups. (**c**) A- DP computed combining A-T- and A-T + groups. (**d**) 1D scores comparison per group. (**e**) Scores computed with A + DP (**f**) Scores computed with A- DP. * indicates significant *p*-values and corresponds post-hoc tests following significant Kruskal–Wallis test. *, 0.01 < *p* ≤ 0.05; **, 0.001 < *p* ≤ 0.01; ***, 0.0001 < *p* ≤ 0.001; ****, *p* ≤ 0.0001. Abbreviations: A, amyloid; T, tau; DP, disease pattern; PC, principal component

*1D analysis:* A combination of the first five PCs explained 50.1% of the total variance (PC1: 20.0%, PC2: 12.8%, PC3: 8.8%, PC4: 4.8%, PC5: 3.7%). The first PC (Fig. 3a) was characterized by predominantly negative values in the cerebral WM and positive values in cortical regions typically affected by AD, including the precuneus, parietotemporal, and frontolateral cortices.

*2D analysis – A* + *DP:* The first five PCs accounted for 51.2% of the variance (PC1: 22.4%, PC2: 11.1%, PC3: 8.6%, PC4: 5.3%, PC5: 3.8%). The A + disease pattern (Fig. 3b) was derived from a weighted linear combination of the first three PCs, with respective weights of 0.919, 0.146, and 0.366. This pattern exhibited an increased signal in cortical regions, particularly in the precuneus, parietotemporal, and frontolateral areas.

*2D analysis – A − DP:* A combination of the first six PCs explained 53.3% of the variance (PC1: 23.7%, PC2: 11.3%, PC3: 6.9%, PC4: 4.1%, PC5: 3.7%, PC6: 3.5%). In this case, the third component alone was identified as the most effective discriminant between groups. The resulting DP (Fig. 3c) was characterized by a scattered increased signal across both cortical and subcortical regions, without clear localization to specific anatomical areas.

*SSM/PCA scores* (Fig. 3d–f): Examining PC1 and the A + DP scores, the largest differences were observed between A + T + and A − T −, as well as between A + T + and A + T − (*p* < 0.01 for all comparisons, corrected for multiple testing). A − T − also differed significantly from both A − T +. No significant difference was observed between A − T + and A + T + for the A − DP scores, nor between A − T − and A + T − for either DPs. Detailed results of the covariate analyses are reported in Supplementary Tables 1–2 and Supplementary Figure S5, while Spearman correlations between the scores and CL/global tau SUVR are shown in Supplementary Figure S6.

### Amyloid status prediction

**Fig. 4:**
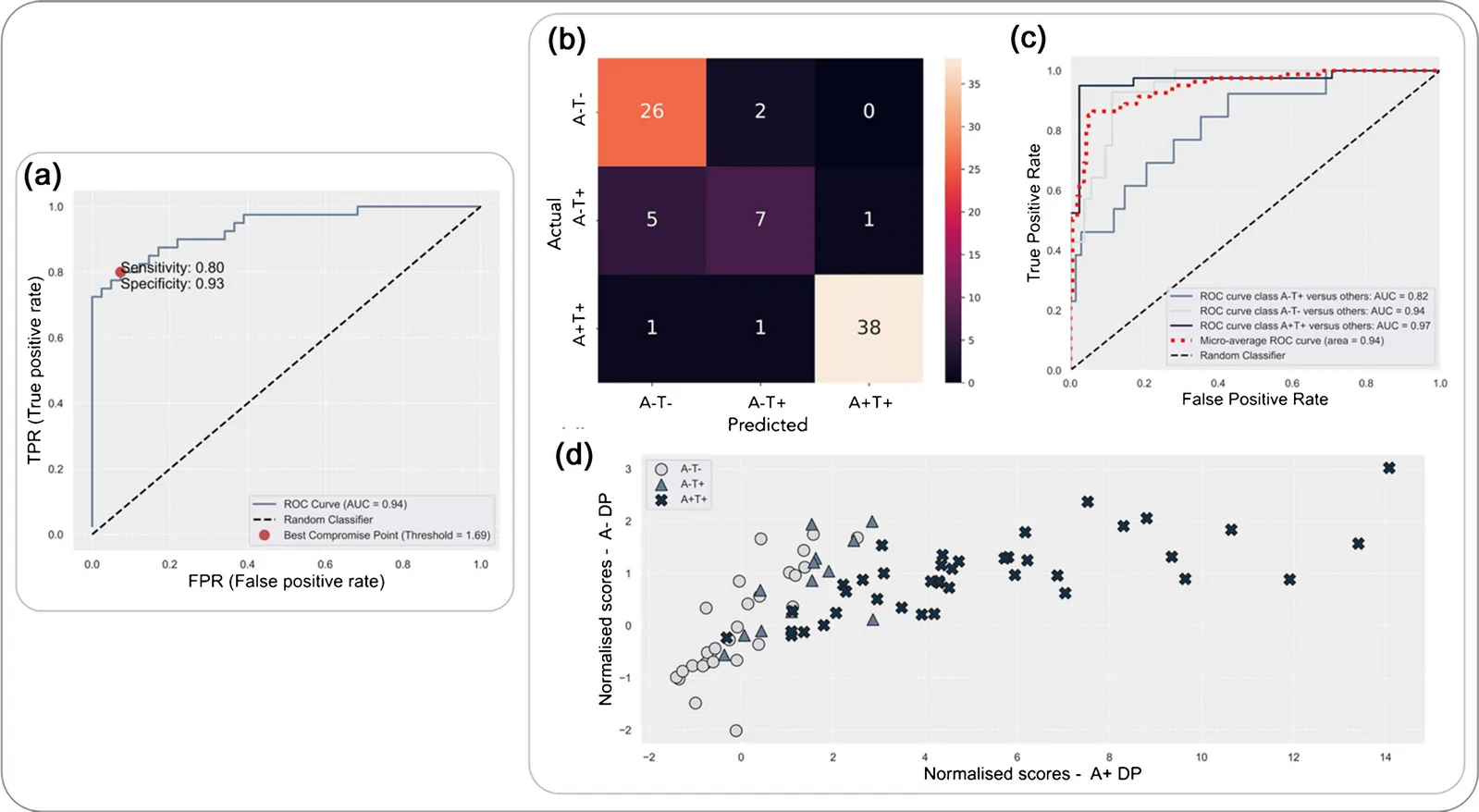
(**a**) 1D binary ROC curve. The optimal balance point was highlighted in red. (**b**) Confusion matrix of the MLP model. (**c**) Multi-class ROC curve of the MLP model. (**d**) Normalised scores in a 2D plane. Abbreviations: A, amyloid; T, tau; AUC, area under the curve; DP, disease pattern; ROC, receiver operating characteristic curve

*1D analysis*: The expression of the PC1 predicted amyloid status with a sensitivity of 0.80 and a specificity of 0.93 (area under the curve (AUC) = 0.94; Fig. 4a). The optimal classification threshold identified using Youden’s method was 1.69 (sensitivity = 0.80, specificity = 0.93).

*2D analysis*: Among the three classifiers, the MLP achieved the highest overall performance (balanced accuracy = 0.81, weighted‑F1 = 0.87, macro‑F1 = 0.81). SVM followed (balanced accuracy = 0.77, weighted‑F1 = 0.84, macro‑F1 = 0.79). In contrast, the RF model yielded lower scores across all metrics (balanced accuracy = 0.65, weighted‑F1 = 0.74, macro‑F1 = 0.66). Figures 4b and 4c display the confusion matrix and the multi-class ROC curve of the best-performing model. Confusion matrices and ROC curves for all models are provided in Supplementary Figure S7, while the sensitivity analysis for class imbalance in presented in Supplementary Figure S8. All models maintained relatively stable performance across iterations, with median balanced accuracy values of approximately 0.72 for MLP, 0.69 for SVM, and 0.64 for RF. Figure 4d illustrates the distribution of normalized scores in a two-dimensional space.

### Correlation between SSM/PCA scores and plasma biomarkers

**Fig. 5:**
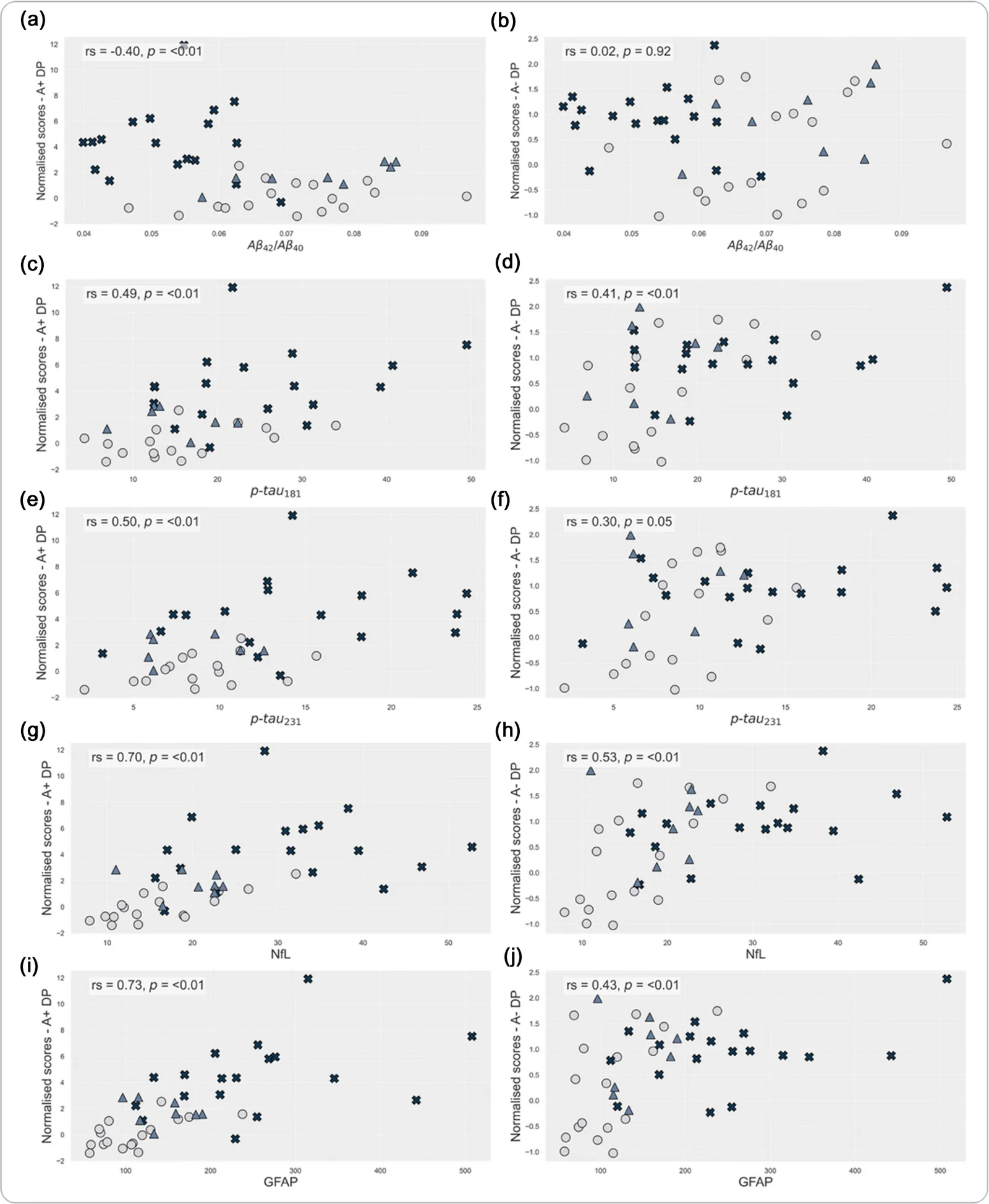
Spearman correlation between SSM/PCA scores and plasma biomarkers. Plasma biomarker values are displayed on *x*-axis and DP scores on *y*-axis. First column shows the correlation with the scores computed with A + DP, and second column with scores computed with the A- DP. (**a** and **b**) Aβ_42_/Aβ_40_, (**c** and **d**) p-tau_181_, (**e** and **f**) p-tau_231_, (**g** and **h**) NfL, (**i** and **j**) GFAP. Light grey dots, A-T-; light blue triangles, A-T + triangles; dark blue crosses, A + T +. Abbreviations: A, amyloid; DP, disease pattern; rs, Spearman correlation coefficient; p-tau, phosphorylated tau; NfL, neurofilament light; GFAP, glial fibrillary acidic protein

Figure 5 illustrates the correlations between SSM/PCA-derived scores and plasma biomarkers. Significant correlations were observed across all comparisons (*p* < 0.01). No significant correlations were found between the A − DP scores and either Aβ_42_/Aβ_40_ (rs = 0.02, *p* = 0.92) or p-tau_231_ (rs = 0.30, *p* = 0.05).

## Discussion

Clinical diagnostic protocols for AD commonly combine A- and T-PET imaging to distinguish AD from other neurodegenerative conditions. This study investigated whether [^18^F]flortaucipir DPs derived using SSM/PCA, could differentiate A + from A − profiles, potentially reducing the need for dedicated A-PET. This analysis was supported by Spearman correlations between SSM/PCA-derived scores and plasma biomarkers of AD pathology.

Prior to SSM/PCA, group differences in imaging and plasma were assessed using the Kruskal–Wallis test. Global [^18^F]flortaucipir SUVR differences were consistent with expectations, showing higher signal in the A + T + group, in line with the tracer’s specificity for AD pathology. As anticipated, signal intensity was generally lower in A–T + subjects, consistent with previous reports in non‑AD tauopathies [11, 23], and minimal in A–T– controls. Reduced Aβ_42_/Aβ_40_ levels in the A + T + group are consistent with impaired amyloid clearance mechanisms, which may involve plaque accumulation, alterations in the glymphatic system, or blood–brain barrier dysfunction. The absence of significant differences in plasma Aβ_42_/Aβ_40_ between the A–T– and A–T + groups confirmed the validity of the group stratification and further supported the correlation between A-PET findings and plasma Aβ_42_/Aβ_40_ levels [27]. Both p-tau biomarkers distinguished the A + T + group from A–T– and A–T +, reinforcing their specificity for AD pathology [27, 28] and supporting evidence that elevated p-tau levels rise concurrently with early amyloid aggregation [7]. Neuronal injury and neurodegeneration are central in AD, but NfL can be elevated in several non-AD conditions [28]. NfL levels were significantly higher in A + T + subjects, consistent with ongoing neurodegeneration, whereas no difference between A–T– and A–T + may reflect milder pathology in the latter. GFAP, an inflammatory marker of reactive astrogliosis, is known to increase in amyloid-positive individuals regardless of tau status [18, 29]. Consistent with this, our findings showed higher GFAP in the A + T + group compared to A − groups, with no difference between A–T– and A–T +.

SSM/PCA identified two distinct DPs. The A + DP exhibited a clear cortical distribution, with prominent tracer uptake in the precuneus, parietotemporal, and frontolateral regions. In contrast, the A − DP showed a more diffuse and less intense signal, primarily involving subcortical WM and grey matter (GM) regions. Score distributions revealed a broader dynamic range for the A + DP, facilitating clearer separation between groups. In contrast, scores derived from the A − DP were confined to a narrower interval, with greater overlap across subjects. Among the tested classifiers, the MLP model achieved the highest balanced accuracy (0.81), although good classification was obtained with all methods pointing towards the value of DPs to distinguish between different amyloid load. Overall, all models confirmed the strong predictive value of the A + DP for AD pathology, whereas the A − DP demonstrated limited discriminative power for non-AD condition. When evaluated in A + T − subjects, both DPs showed scores similar than those of A − T − individuals and lower than those of A + T + subjects. No statistical difference with A − T + individuals could be attributed to the limited expressiveness of the A − DP. Although the DPs were derived from tau PET images, these results provide further evidence regarding the biological drivers of the identified patterns, indicating that their expression is primarily linked to tau-related processes rather than amyloid positivity alone. Additional analyses adjusting for demographic and clinical covariates showed that, while MMSE and gender account for part of the variability in the scores, they do not drive the overall structure of the observed effects. This suggests that the PCA-derived patterns capture spatial variability in tau deposition beyond general disease severity. From a clinical perspective, this supports the specificity of the patterns and their potential utility for interpreting tau PET scans. Consequently, the apparent ability of the model to identify A + subjects is not directly driven by amyloid deposition itself, but rather by the high probability that T + subjects are also A + in our cohort.

Both DPs showed significant correlations with plasma biomarkers, except for Aβ_42_/Aβ_40_ and p-tau_231_, which did not correlate significantly with scores derived from the A − DP.

The spatial distribution of the A + DP closely aligned with tau pathology corresponding to Braak stage IV or higher, with tracer uptake observed in regions typically affected in AD [10]. This pattern was also evident in the univariate t-test analysis; however, the SSM/PCA approach offered additional advantages by capturing inter-voxel covariance and enabling the computation of subject scores for individual assessment. Notably, while comparisons of CL values across groups did not reveal a significant difference between A-T- and A-T + subjects, DP scores were able to distinguish these groups, highlighting the added value of the multivariate SSM/PCA approach.The robustness and disease specificity of the A + DP were further supported by its significant correlations with all plasma biomarkers and its superior discriminative performance across classification models. Nevertheless, these significant correlations may also reflect the high specificity of the included plasma biomarkers for AD pathology. The A − DP exhibited a distinct signal, consistent with previous findings reporting subcortical white and gray matter binding with generally lower intensity in FTLD [25, 30]. However, its diffuse distribution and lack of a clear anatomical focus may reflect the heterogeneity within the A − T + group, which likely encompasses a spectrum of non-AD tauopathies − such as progressive supranuclear palsy, corticobasal degeneration, FTLD, and Pick’s disease − each associated with distinct tau isoforms (4R vs 3R) and characteristic topographic patterns [11, 30–33]. Importantly, this group was defined solely based on the absence of amyloid pathology, without further etiological stratification, leaving the underlying pathologies undefined. Such heterogeneity likely contributes to the reduced specificity and limited discriminative power of the A − DP, as observed in the classification step. Moreover, it currently remains unclear whether [^1^⁸F]flortaucipir binding in non-AD pathologies reflects a specific affinity for tau isoforms typical of these diseases, or instead a nonspecific uptake due to co-pathologies occurring in overlapping regions [34, 35].

A study published by Hammes and colleagues in 2020 [14], found that [^18^F]flortaucipir can effectively discriminate between A + and A − neuro-pathologies. However, their study focused on a specific non-AD population, which could potentially introduce a population selection bias. In memory clinics, non-AD conditions are a rare form of neurodegeneration, making it challenging to find populated groups with specific non-AD forms when investigating AD biomarkers. Although the DPs identified in this work were comparable to those found by Hammes and colleagues, their promising classification results may be driven by the specificity of their A-T + group being focused on FTLD. This specificity may not always be present in clinical practice, highlighting the relevance and the replicability of this study.

In summary, this work supports the use of [^1^⁸F]flortaucipir-derived DPs as a valuable tool to streamline the clinical diagnostic workflow in suspected AD cases. This approach may offer both economic and safety advantages. However, for all other patients, the standard protocol—including both A- and T-PET imaging—remains the recommended strategy [38, 39].

This study has few limitations. First, A + T + subjects accounted for 50% of the study population, and a strong similarity is present between the PC1 and the A + DP, suggesting that the A + T + group largely drives the variability within the dataset. This class imbalance requires careful interpretation and the use of performance metrics that are robust to unequal class sizes. However, the sensitivity analysis indicated that, even after balancing the classes through random undersampling of the larger classes, all models maintained relatively stable performance across iterations. Second, due to the relatively small sample size, particularly after dividing participants into three groups, the stability and generalizability of the machine learning analyses may be limited. Although cross-validation predictions were used to reduce overfitting, hyperparameter tuning and model evaluation were performed on the same dataset. Therefore, while this approach maximizes the use of available data, some degree of optimistic bias cannot be excluded, and external validation in an independent cohort will be necessary to confirm generalizability. Finally, analyses involving plasma biomarkers were limited by incomplete data availability since only 43 out of the 81 participants underwent plasma sampling, reducing the sample size for correlation analyses.

Future research could broaden the AT framework by incorporating additional biomarkers, such as neurodegenerative or inflammatory markers, and by considering cognitive stage to further stratify subgroups (e.g., distinguishing MCI from dementia within A + T +). The predictive phase could be enhanced by including demographic variables (age, sex, and education), clinical measures (neuropsychological testing), and imaging-derived features, while etiological information may help explain the heterogeneity of the A–T + group. Validation of DPs remains essential: although LOOCV was applied, independent cohort testing and evaluation in new patients are needed. Finally, recent revisions of the ATN framework have leveraged imaging to capture topographic information and classify tau pathology severity into three disease stages based on affected regions [7]. Since no automated algorithm currently exists for this subdivision, SSM/PCA could be explored as a tool to classify individuals into disease stages and improve prognostic accuracy.

## Conclusion

To conclude, this analysis indicated that [^18^F]flortaucipir-PET DPs derived from SSM/PCA provide information on both A and T status, supporting its potential use as first and possibly sole examination in the investigation of suspected AD. For other clinical cases, A-PET scans are still recommended. These results demonstrate the effectiveness of SSM/PCA motivating to be further explored in the clinic and research settings.

## Supplementary Information

Below is the link to the electronic supplementary material.Supplementary file1 (DOCX 2999 KB)

## Data Availability

Datasets generated during and/or analysed during the current study are available from the corresponding author upon reasonable request.
