# Supplementary material for: Investigating AD and non-AD [18F]flortaucipir distribution patterns in a memory clinic cohort
Source: Eur J Nucl Med Mol Imaging. 2026 Jun 15;53(11):6238–51. doi: 10.1007/s00259-026-07995-z (PMC13526141; doi:10.1007/s00259-026-07995-z)
Supplement: Supplementary file 1 [file 259_2026_7995_MOESM1_ESM.docx]

Investigating AD and non-AD ^18^F-Flortaucipir distribution patterns in a memory clinic cohort

Francesco Piva^1^, Cecilia Boccalini^2^, Nicholas J. Ashton^3,4,5,6^, Henrik Zetterberg^4,7,8,9,10,11^, Kaj

Blennow^4,9,12,13^, Giovanni B. Frisoni^14,15^, Valentina Garibotto^2,16,17^, Débora Elisa Peretti^2^

1	Department of Information Engineering, University of Padua, Padua, Italy

2	Laboratory of Neuroimaging and Innovative Molecular Tracers, University of Geneva, Geneva, Switzerland

3	Centre for Age-Related Medicine, Stavanger University Hospital, Stravanger, Norway

4	Department of Psychiatry and Neurochemistry, The Sahlgrenska Academy at the University of Gothenburg, Mölndal, Sweden

5	Institute of Psychiatry, Psychology & Neuroscience, King-s College London, London, UK

6	NIR Biomedical Research Centre for Mental Health & Biomedical Research Unit for Dementia at South London & Maudsley NHS Foundation, London, UK

7	UK Dementia Research Institute, University College London, London, UK

8	Department of Neurodegenerative Diseases, University College London, London, UK

9	Clinical Neurochemistry Laboratory, Sahlgrenska University Hospital, Mölndal, Sweden

10	Department of Pathology and Laboratory Medicine, University of Wisconsin School of Medicine and Public Health, Madison, WI, USA

11	Wisconsin Alzheimer’s Disease Research Centre, University of Wisconsin-Madison, Madison, USA

12	Paris Brain Institute, Sorbonne University, Paris, France

13	Neurodegenerative Disorder Research Centre, University of Wisconsin-Madison, Madison, USA

14	Laboratory of Neuroimaging of Aging, University of Geneva, Geneva, Switzerland

15 Geneva Memory Center, Geneva University Hospitals, Geneva, Switzerland

16	Division of Nuclear Medicine and Molecular Imaging, Geneva University Hospitals, Geneva, Switzerland

17 Centre for Biomedical Imaging, University of Geneva, Geneva, Switzerland

Corresponding author: Débora Elisa Peretti

Rue Gabrielle-Perret-Gentil 4, 1205, Geneva, Switzerland

[Debora.Peretti@unige.ch](mailto:Debora.Peretti@unige.ch)

**Supplementary Fig. 1** Histograms showing the distribution of global amyloid burden expressed in Centiloid (CL) and tau PET SUVR values for each A/T group (A−T−, A−T+, A+T−, A+T+). Vertical dashed lines indicate the thresholds used to define amyloid and tau positivity (19 CL for amyloid and SUVR = 1.24 for tau). Abbreviations: A, amyloid; T, tau; CU, cognitively unimpaired; MCI, mild cognitive impairment; SUVR, standardized uptake value ratio

**Supplementary Fig. 2** Regional SUVR comparison. (a) regions involved in the firsts three Braak stages: hippocampus, parahippocampus, fusiform gyrus, lingual gyrus, and amygdala. (b) regions involved in Braak stage IV: inferior and middle temporal lobe, temporal pole, insula, and post cingulate (c) regions involved in Braak stage V: frontal lobe, parietal lobe, occipital lobe, superior temporal lobe, precuneus, caudate nucleus, and putamen (d) regions involved in Braak stage VI: precentral and postcentral gyrus, paracentral lobule, and cuneus. * indicates significant *p*-values and corresponds post-hoc tests following significant Kruskal-Wallis test. *, 0.01 < *p* ≤ 0.05; **, 0.001 < *p* ≤ 0.01; ***, 0.0001 < *p* ≤ 0.001; ****, *p* ≤ 0.0001

**Supplementary Fig. 3** Centiloid (CL) comparison between groups. * indicates significant p-values and corresponds post-hoc tests following significant Kruskal-Wallis test. *, 0.01<p$\leq$0.05; **, 0.001<p$\leq$0.01; ***, 0.0001<p$\leq$0.001; ****, p$\leq$0.0001.

**Supplementary Fig. 4** Two sample t-test statistical parametric mapping (SPM) axial maps rendered on to a normalized T1w-MRI image, shows areas of significant differences. The colour intensity in each image corresponds to the magnitude of the statistical difference observed. Thresholded SPM images (p < 0.001, FWE-corrected at the cluster level) are overimposed on a standard CH2 brain with cerebellum surface using BrainNet Viewer Comparison: A+T+ < A-T+. Abbreviations: A, amyloid, T, tau.

**Supplementary Table. 1** Results of the linear models assessing the effects of group and covariates on A+ DP scores. The table reports regression coefficients (β), standard errors (SE), t-statistics, and p-values for each predictor.

| Variable | Coefficient ($\beta)$ | Std.Error | t-value | p-value |
| --- | --- | --- | --- | --- |
| Intercept | 11.16 | 2.46 | 5.16 | < 0.01 |
| C(profile) A-T+ | -2.33 | 0.65 | -3.60 | <0.01 |
| C(profile) A-T+ | 0.92 | 0.71 | 1.29 | 0.20 |
| C(profile) A+T+ | 4.24 | 0.59 | 7.19 | < 0.01 |
| Gender (male) | -1.43 | 0.44 | -3.30 | < 0.01 |
| age | -0.05 | 0.03 | -1.80 | 0.074 |
| education | 0.07 | 0.06 | 1.24 | 0.22 |
| MMSE | -0.27 | 0.06 | -4.65 | < 0.01 |

**Supplementary Table. 2** Results of the linear models assessing the effects of group and covariates on A- DP scores. The table reports regression coefficients (β), standard errors (SE), t-statistics, and p-values for each predictor.

| Variable | Coefficient ($\beta)$ | Std.Error | t-value | p-value |
| --- | --- | --- | --- | --- |
| Intercept | 0.83 | 0.85 | 0.98 | 0.33 |
| C(profile) A+T- | -1.12 | 0.24 | -4.64 | <0.01 |
| C(profile) A-T+ | 0.59 | 0.27 | 2.23 | < 0.05 |
| C(profile) A+T+ | 0.77 | 0.22 | 3.50 | < 0.01 |
| Gender (male) | -0.72 | 0.16 | -4.44 | < 0.01 |
| age | 0.003 | 0.01 | 0.22 | 0.83 |
| education | 0.03 | 0.02 | 1.21 | 0.23 |
| MMSE | -0.03 | 0.02 | -1.56 | 0.12 |

x

**Supplementary Fig. 5** Normalised SSM/PCA scores (adjusted) computed with A+ DP (left), scores (adjusted) computed with A- DP (right). * indicates significant *p*-values and corresponds post-hoc tests following significant Kruskal-Wallis test. *, 0.01<*p*≤0.05; **, 0.001<*p*≤0.01; ***, 0.0001<*p*≤0.001; ****, *p*≤0.0001. Abbreviations: A, amyloid; T, tau; DP, disease pattern; PC, principal component.

**Supplementary Fig. 6.** Spearman correlations between SSM/PCA scores and CL/global tau SUVR. CL and global tau SUVR values are shown on the x-axis, and DP scores on the y-axis. The first column displays correlations with scores computed using the A+ DP, and the second column with scores computed using the A- DP. (a–b) Light grey dots: A-T-; light blue triangles: A-T+; dark blue crosses: A+T+. Abbreviations: CL, centiloid; SUVR, standardized uptake value ratio.

**Supplementary Fig. 7** Comparison of confusion matrices (CM) and Receiver Operating Characteristic (ROC) Curves ordered from worst to best. (a) CM of Random Forest (b) CM of Support Vector Machine (c) CM of Multilayer Perceptron (d) ROC curve of Random Forest (e) ROC curve of Support Vector Machine (f) ROC curve of Multilayer Perceptron. Abbreviations: A, amyloid; T, tau

**Supplementary Fig. 8** Sensitivity analysis comparing the performance of three classification models—Random Forest (RF), Support Vector Machine (SVM), and Multilayer Perceptron (MLP). Model performance was evaluated across 30 repeated undersampling iterations to mitigate class imbalance. For each iteration, the larger classes were randomly undersampled to match the size of the smallest class. Performance metrics include balanced accuracy, weighted F1-score, and macro F1-score, summarizing the stability and variability of each model across resampling runs.
